# Microbiota-Meditated Immunity Abnormalities Facilitate Hepatitis B Virus Co-Infection in People Living With HIV: A Review

**DOI:** 10.3389/fimmu.2021.755890

**Published:** 2022-01-06

**Authors:** Jing Ouyang, Silvere D. Zaongo, Xue Zhang, Miaomiao Qi, Aizhen Hu, Hao Wu, Yaokai Chen

**Affiliations:** ^1^ Division of Infectious Diseases, Chongqing Public Health Medical Center, Chongqing, China; ^2^ Department of Infectious Diseases, You’an Hospital, Capital Medical University, Beijing, China

**Keywords:** HIV infection, hepatitis B virus co-infection, gut epithelial damage, microbiota, immunity

## Abstract

Hepatitis B virus (HBV) co-infection is fairly common in people living with HIV (PLWH) and affects millions of people worldwide. Identical transmission routes and HIV-induced immune suppression have been assumed to be the main factors contributing to this phenomenon. Moreover, convergent evidence has shown that people co-infected with HIV and HBV are more likely to have long-term serious medical problems, suffer more from liver-related diseases, and have higher mortality rates, compared to individuals infected exclusively by either HIV or HBV. However, the precise mechanisms underlying the comorbid infection of HIV and HBV have not been fully elucidated. In recent times, the human gastrointestinal microbiome is progressively being recognized as playing a pivotal role in modulating immune function, and is likely to also contribute significantly to critical processes involving systemic inflammation. Both antiretroviral therapy (ART)-naïve HIV-infected subjects and ART-treated individuals are now known to be characterized by having gut microbiomic dysbiosis, which is associated with a damaged intestinal barrier, impaired mucosal immunological functioning, increased microbial translocation, and long-term immune activation. Altered microbiota-related products in PLWH, such as lipopolysaccharide (LPS) and short-chain fatty acids (SCFA), have been associated with the development of leaky gut syndrome, favoring microbial translocation, which in turn has been associated with a chronically activated underlying host immune response and hence the facilitated pathogenesis of HBV infection. Herein, we critically review the interplay among gut microbiota, immunity, and HIV and HBV infection, thus laying down the groundwork with respect to the future development of effective strategies to efficiently restore normally diversified gut microbiota in PLWH with a dysregulated gut microbiome, and thus potentially reduce the prevalence of HBV infection in this population.

## Introduction

Human immunodeficiency virus (HIV) infection and chronic hepatitis B virus (HBV) infection are major global public health concerns, and prior studies have shown that interplay can occur between these viruses (1–3). HIV is a pathogen that causes systemic CD4+ T-cell destruction and results in impaired cell-mediated immunity, which leads to the development of various opportunistic infections and non-AIDS comorbidities (4). Infection by HBV is a major cause of liver diseases, including chronic hepatitis B, liver cirrhosis, and hepatocellular carcinoma. Most healthy adults infected with HBV are able to neutralize the virus during the acute phase of infection *via* immunological mechanisms. However, among infants and young children, chronic HBV infections frequently eventuate because of their immature immune systems (5). In people living with HIV (PLWH), co-infection with HBV is fairly common and contributes significantly to morbidity and mortality (1, 3, 6–8). There is a significant increase in HBV DNA levels, higher hepatitis B e antigen (HBeAg) fraction, lower CD4+ T-cell counts, and poorer liver and coagulation functions among HBV/HIV co-infected individuals, compared to mono-infected individuals (7). Immune dysfunction caused by HIV enhances the likelihood of HBV persistence, and hepatotoxicity associated with anti-HIV therapy may exacerbate the liver diseases associated with HBV persistence (9).

Multiple factors have been considered as potential contributors to the high prevalence of HBV in PLWH, such as identical transmission routes of the individual HIV and HBV viruses, HIV-induced immune suppression, poor HBV vaccination response and reduced liver function. Aside from these factors, recent research observations indicate that microbiota-maintained immunity is also involved in HIV and HBV co-infection (10–14). The human body is a host to over 10^14^ micro-organisms, including bacteria, fungi, archaea, viruses, and eukaryotic microbes, which is 1-10 times greater than the number of endogenous host cells in humans (15, 16). In recent times, increasing evidence has demonstrated that microbiome composition, diversity, and microbial products play a pivotal role in maintaining good health through several metabolic and immune pathways. Microbiota dysbiosis has been shown to be involved in the pathogenesis and progression of numerous inflammatory- and immune-related diseases, including inflammatory bowel diseases, cancer, HBV infection, and HIV infection. In this review, we critically discuss advances in the understanding of the underlying causes of the high prevalence of HBV infection in PLWH, and hypothesize how microbiota-meditated immunity abnormalities in PLWH may facilitate hepatitis B virus co-infection.

## HIV Infection Leads to Immune Impairment and Long-Term Systemic Inflammation

CD4+ T-cells play multiple roles in orchestrating the overall response to viral infections by coordinating the diverse components of the immune system. Their functions include helping in B-cell-mediated high affinity antibody production, enhancement of CD8+ T-cell expansion, function, and memory, and establishment of cellular and humoral antigen-specific immunity, which is the cornerstone of long-term protection from a diverse range of microbial infections and is the fundamental principle behind the effectiveness of most vaccines (17, 18). However, HIV can specifically bind to T-lymphocytes expressing the CD4+ receptor, and is able to thus interact with cellular co-receptors (e.g., chemokine receptor CCR5 or CXCR4), and subsequently infect and destroy these T-lymphocytes (19, 20). By progressively destroying HIV-infected CD4+ T-cells, HIV infection induces profound cellular immunodeficiency and, hence, an inability of the immune system to function in a competent manner.

The introduction of combined antiretroviral therapy (ART) for the treatment of HIV infection has resulted in persistent suppression of HIV replication and recovery of CD4+ T-cell counts in the majority of patients, thus leading to large declines in both mortality and morbidity in PLWH. However, the degree of immune recovery achieved under ART varies greatly between individuals. There is a fraction (between 10% to 40%) of HIV-infected patients on ART whose absolute CD4+ T-cell counts remain less than 200 cells/μl despite suppression of HIV replication after even years of ART (21–24). These patients are considered to be immunological discordants or non-responders (INRs), in contrast to immunological responders (IRs) whose CD4+ T-cell counts reach 200 cells/μl or over with suppression of HIV replication (25). Compared with IRs, INRs have a poorer overall immune response and a higher risk of clinical progression to AIDS (24–26).

IRs experience persistent chronic immune activation and inflammation, leading to an increased risk of the development of non-AIDS co-morbidities, such as the metabolic syndrome and certain cancers. Several factors contribute to this ongoing immunological and inflammatory state, including persistent antigenic stimulation by the low residual viremia, gut microbial translocation due to a leaky gut and microbiota dysbiosis, thymic dysfunction, other opportunistic co-infection, and ART toxicity (27–29). During the early stages of HIV infection, intestinal CD4+ T-cells are massively depleted as these cells express high levels of the CCR5 co-receptor, permitting the entry of HIV virions into these cells, followed by eventual destruction of the cells and, in the gut, disruption of gut mucosal epithelial barrier integrity, and this may not fully resolve even with early ART initiation (30–32). The disruption of gut homeostasis leads to increased translocation of microbial products such as microbial DNA, bacterial lipopolysaccharide (LPS), and the fungal polysaccharide, (1→3)-β-D-Glucan (BDG), from the gut to the portal and systemic circulation, thus promoting and sustaining chronic immune activation (33, 34). Mehraj et al., reported that plasma BDG levels were elevated during early and chronic HIV infection and persisted despite long-term ART, and had an inverse correlation with CD4+ T-cell counts, Dectin-1 on monocytes, and NKp30 expression on NK cells (34). The varying and unpredictable degrees of immune impairment and the chronic underlying systemic inflammation induced by HIV have evolved into issues of significant importance for PLWH as well as for HIV researchers and clinicians in the prevailing ART era.

## High HBV Infection Prevalence IN PLWH

Globally, the latest data from the World Health Organization (WHO) indicates that 2.7 million people are co-infected with HIV and HBV (3). In concordance with these figures, a global meta-analysis conducted in 2020, which included 475 studies done in 80 countries, observed that the prevalence of HIV-HBV co-infection in PLWH was 7.6%, equating to 2.7 million HIV-HBV co-infections (35). An epidemiological study in the US conducted over approximately 20 years (1989-2007) observed that HBV co-infection is relatively common in PLWH. Specifically, 1078 (39%) of 2769 PLWH had comorbid HBV infection, among which 117 subjects had chronic hepatitis B (36). The incidence of HBV infection following HIV diagnosis has decreased dramatically from 4.0/100 person-years in the pre-ART era to 1.1/100 person-years in the current ART era, but has remained constant since the beginning of the 21^st^ century (36). A systematic review conducted in Europe in 2019 observed that HBV infection prevalence ranged between 2.9% and 43.4% in PLWH (37). Konopnicki et al., reported that the prevalence of HBV co-infection in PLWH was more than 100-fold the prevalence seen in the general population in the same region, and that chronic HBV infection significantly increased liver-related mortality in HIV-infected patients (38). A meta-analysis in China found that pooled HBV prevalence in PLWH was 13.7% (95% CI 12.3-15.3%), with variations found with respect to age and geographic region (39). Overall, the findings of these studies indicate that HBV co-infection is appreciably prevalent in PLWH.

## Main Factors Associated With HBV Infection in PLWH

Several factors contribute to the increased prevalence of HBV co-infection in PLWH. HIV and HBV infection share identical transmission routes. Both are transmitted from person to person through semen, blood, or other bodily fluids. Thus, people at risk for HIV infection are also at risk for HBV infection, especially in high-risk groups such as people who have sex without a condom and those who inject recreational drugs (PWID). However, data from recent global meta-analyses are not fully concordant with what has previously been assumed (35, 40). In 2020, one meta-analysis indicated that the prevalence of HBV among PLWH/PWID was slightly higher (11.8% IQR 6.0%-16.9%) than that among PLWH who had not used recreational injectable drugs (6.1% IQR 4.0%-9.9%) (35). Based on 70 studies, the median HBV prevalence among men who have sex with men (MSM) with HIV/AIDS was 6.1% (IQR 5.0%‐9.2%) (35). In the same year as the preceding study was conducted, another global meta-analysis observed that the prevalence of HBV among PWID with HIV/AIDS was 8% (95% CI: 5%-13%). Interestingly however, the prevalence of HBV among female sex workers (FSWs) with HIV/AIDS was found to be remarkably low, at 2% (95% CI: 0%-7%) (40).

Moreover, a patient’s inherent ability to clear HBV is now known to be reduced by HIV-induced immune suppression (41–43). Most HIV-negative individuals have the ability to spontaneously clear HBV infection, while PLWH who are exposed to HBV have an approximately 3-6 fold higher likelihood of developing chronic HBV infection (9). Cohen Stuart et al., reported that occult hepatitis B in PLWH is associated with low CD4+ T-cell counts (42). Compared with HIV-negative and HBV-immune patients, a reduction in HBV-specific CD8+ T-cell responses in HIV/HBV patients was observed in a cross-sectional study (41). The introduction of ART can reconstitute some HBV-specific CD4+ and CD8+ T-cell responses, in association with increased restoration of CD4+ T-cell counts (41).

A poor immunological response to HBV vaccination may be another factor affecting HBV co-infection in PLWH. HBV vaccination is an important strategy for prevention of HBV infection and is highly effective in general, with vaccine response rates greater than 90% (44). However, PLWH, especially those with low CD4+ T-cell counts, have a considerably lower response rate to HBV vaccination of between 18–71%, and more rapid rates of antibody decline after acquisition of protective anti-HBs Ab titers from vaccination, compared to HIV negative individuals (45–49). A study conducted in Taiwan observed that the serological response rate to HBV revaccination in HIV-positive MSM patients was modest and that the antibody titers generated by revaccination was found to wane rapidly, despite initial HBV vaccination in the neonatal period as part of the childhood vaccination schedule. Therefore, reinforced HBV vaccination strategies have been recommended for PLWH (50).

Additionally, other than hepatitis B, PLWH are also more susceptible to other liver diseases, such as non-alcoholic fatty liver disease (NAFLD), fibrosis, and cirrhosis. Compared with HIV negative individuals, HIV mono-infected patients have a higher frequency of liver fibrosis, and its prevalence ranges between 11% and 41% (51–55). These liver diseases may be induced either by direct hepatocyte destruction due to HIV, hepatotoxicity from ART drugs, and the heavy burden of microbial products presented to the liver in these patients (56–58). As the largest population of resident macrophages in the liver, Kupffer cells play a key role in liver injury, hepatic inflammation, and clearance of HBV from the liver. The protective effects of Kupffer cells are due to their inherent role as innate immune cells (59–63). However, as these cells also express CD4+, CCR5, and CXCR4, they are vulnerable and can be productively infected by HIV, resulting in a dysregulated hepatic innate immune response, subsequent hepatic inflammation, and fibrosis (56, 64, 65). In addition, specific ART regimens, especially nevirapine-, efavirenz-, and dolutegravir-containing regimens, have been associated with potentially lethal hepatotoxicity (66–70). Polo et al., observed that hepatic damage induced by efavirenz involved acute interference with mitochondria, and similar observations have been made by other investigators (67, 71–73). The hepatic dysfunction associated with HIV infection and ART is likely to further accelerate the progression of HBV infection (63, 74). It has been reported that hepatocytic mitochondrial dysfunction stimulates HBV gene expression through lipogenic transcription factor activation (74).

Furthermore, aside from the preceding pathogenic mechanisms, recent observations suggest that gut microbiota dysbiosis and the leaky gut phenomenon observed during HIV infection are additional factors favoring HBV infection in PLWH.

## Microbiota Dysbiosis and Leaky Gut in PLWH Increase Susceptibility to HBV

### HIV Infection Provokes Gut Microbiota Dysbiosis and Immune System Impairment

Microbial communities residing in the intestines of PLWH have been shown to significantly differ from those not infected with HIV, and this difference manifests independently of age, gender, and sexual practice (75, 76). HIV-1 infection is now recognized to be characterized by microbial dysbiosis, which is associated with a damaged intestinal barrier, impaired mucosal immunological function, increased microbial translocation, and long-term immune activation in PLWH (14, 75–80). According to past research data, gut microbiota dysbiosis in PLWH mainly manifests as changes in microbial diversity, a reduction in symbiotic beneficial bacteria, and an increase in potentially pathogenic bacteria (79, 80). The changes in gut microbiota diversity observed in past studies have not always been found to be consistent, as different studies have involved different populations and in varying disease contexts. Most studies have shown that, in PLWH, the alpha (α-) diversity, which is a common outcome of interest in microbiome research (81), of intestinal microbiota is decreased (82–84), while other studies have found that the α-diversity increased or remained unchanged (85, 86). With the intention of settling the doubt created by these divergent observations, a recent meta-analysis examined 22 studies and concluded that HIV-positive status was associated with a significant decrease in measures of α-diversity (87). In addition, it has been reported that in PLWH, gut microbiota alterations are closely associated with immune dysfunction, and lower bacterial α-diversity correlates with lower CD4+ T-cell counts and higher markers of microbial translocation and monocyte activation (88).

It has been shown, on one hand, that the abundance of “beneficial” bacteria, including *Akkermansia muciniphila*, *Bacteroides, Faecalis, Bacteroides vulvae, Diplococcus*, and *Arbuscular Roseus*, are reduced in PLWH compared with HIV negative individuals (77, 83, 89, 90). On the other hand, however, a higher proportion of potentially pathogenic microorganisms such as *Proteus, Enterococcus, Klebsiella*, *Shigella*, and *Streptococcus* have been reported in PLWH (83, 89). The specific case of *A. muciniphila* provides a clearer picture of the impact of HIV infection on the gut and on an individual’s immune homeostasis. Past investigations have recognized that *A. muciniphila* supports intestinal mucosal homeostasis by modulating mucus thickness (91). *A. muciniphila* has thus been studied by our research team with respect to its intestinal symbiotic interactions (76), and has emerged as a potential “sentinel of the gut” due to its relatively recently recognized beneficial effects, which include (i) stimulation of gut mucin production, (ii) improvement of enterocyte monolayer integrity, (iii) counteracting of inflammation, and (iv) induction of intestinal adaptive immune responses (92–95). A significantly lower intestinal abundance of *A. muciniphila* has been observed in PLWH, regardless of whether or not on ART and of prevailing CD4+ T- cell counts or viral loads, compared to healthy controls (77, 90).

**Figure 1:**
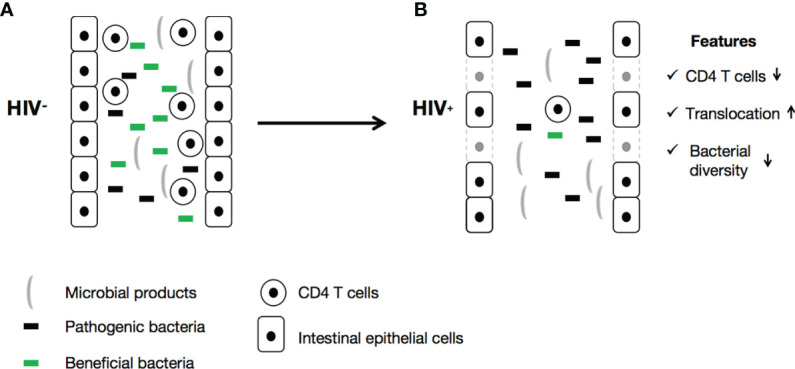
HIV is responsible for gut microbiota dysbiosis and the onset of leaky gut syndrome.

The changes to gut microbiota in HIV-infected patients can impact their immune function *via* many mechanisms. T-helper 17 (Th17) cytokines, secreted by CD4+ T-cells, CD8+ T-cells, gamma delta (γδ) T-cells, natural killer T-cells (NKT), and natural killer (NK) cells, play a vital role in modulating adaptive immune responses through different cellular signaling pathways (96). These cytokines promote mucosal barrier function through enhancement of the epithelial release of antimicrobial peptides, induction of mucus production, and promotion of wound healing (75, 97). HIV infection disrupts the intestinal epithelial barrier (**Figure 1**), resulting in intestinal cell apoptosis and disruption of tight junctions, which leads to the leaky gut syndrome, which is manifested by increased microbial translocation. Additionally, depletion of CD4+ Th17 cells and the high levels of cytokines induced by HIV leads to persistent activation of immune cells and the production of increased inflammatory cytokines, including IL-1β, IL-6, and TNF-α (89, 98).

Changes in microbiota composition may influence the development, differentiation, and maturation of immune cells. On the one hand, immune cells are known to be primarily generated by bone marrow hematopoietic stem cells. The decreased degree of complexity of gut microbiota, on the other hand, has been associated with a reduction in myeloid cells in the bone marrow and a delay in the clearance of systemic bacterial infections (99). This implies that the formation of hematopoietic cells in bone marrow occurs under the influence of the gut microbiome. As such, it has been reported that the gut microbiota is involved at every step of bone marrow cell development, influencing (i) the migration and gene expression of tissue-resident myeloid cells, as well as (ii) the production of bone marrow and circulating granulocytes by modulating local metabolites and tissue-specific mediators (100). In addition to influencing the development of the myeloid arm in the congenital immune system, gut microbiota has been linked to the regulation of innate lymphocytes (ILCs). ILCs represent a group of innate immune cell that includes both cytotoxic (NK) and non-cytotoxic subpopulations (ILC1-3) (101). Sawa et al., have concluded that gut microbiological signals undoubtedly influence normal ILC maturation and acquisition of function (102). For example, intestinal bacteria can influence ILC3 activity either (i) *via* a direct signal through the pattern recognition receptor (PRR) on ILC3s or (ii) *via* a regulation of intestinal myeloid cells and epithelial cells, which in turn affects the function of ILC3s.

HIV infection elicits changes in enteric microbiota and alters microbial metabolite production. The alteration of microbial metabolites causes subsequent immune deficiency and inflammation. Of note, the majority of short-chain fatty-acids (SCFA) are metabolites produced by intestinal bacteria utilizing dietary fiber fermentation. Among these, butyric acid is an important energy source that contributes to the growth of colonic cells, and plays an important role in maintenance of the integrity of the intestinal epithelium and protection of the intestinal barrier. In addition, SCFA promote the production of host antimicrobial peptides and induce the differentiation of Treg cells, thus effectively inhibiting the proliferation of pro-inflammatory bacteria and reducing the occurrence of chronic inflammation (103). Dillon et al. (104), have observed that the abundance of these SCFA-producing bacteria significantly decreases after HIV infection, leading to reduced total SCFA production, which may promote the activation of intestinal T-lymphocytes and enhance HIV replication. Besides, LPS is an integral component of the cell wall of gram-negative bacteria, and is one of the important markers for microbial translocation. LPS can induce antigen-presenting cells to secrete pro-inflammatory cytokines, such as IL-6, IL-8, and TNF-α. In 2006, Brenchley et al. (33), first demonstrated that increased plasma LPS levels induced systemic immune activation in both HIV-infected and simian immunodeficiency virus-infected rhesus monkey models. To further illustrate the role of metabolites, it is worth noting that Vujkovic-Cvijin et al., have observed that during HIV infection, dysbiosis of gut microbiota correlates with activity of the kynurenine pathway of tryptophan metabolism. These investigators have reported that gut-resident bacteria with capacity to metabolize tryptophan through the kynurenine pathway are enriched in HIV-infected subjects (14). Thus, 3-hydroxyanthranilate, a subproduct of the kynurenine pathway, has been found to accumulate in the gut of HIV positive individuals (103).

The kynurenine pathway is the main pathway for tryptophan metabolism. Excessive kynurenine may affect intestinal mucosal immunity *via* the binding of kynurenine to the aryl hydrocarbon receptor, which in turn can inhibit the differentiation of Th17 cells, leading to intestinal barrier dysfunction, intestinal immune imbalance, and inflammation (75). Subsequent to HIV infection, gut microbiota enhances the degree of tryptophan catabolism to kynurenine, resulting in an increase in local and systemic tryptophan metabolism.

Altogether, the changes subsequent to HIV infection disrupts the gut microbiome, which is likely, in turn, to enhance the susceptibility of an HIV-positive individual to developing HBV infection.

### HIV Infection Favors HBV Infection Establishment

During its lifetime, the human body gradually establishes a stable intestinal flora composition and diversity, which regulates and maintains the body’s health. It is recognized that the intestinal microbiome is under the influence of factors such as the host genetic make-up, host diet, and the gut environment (105–107). As preceding evidence suggests, diseases such as HIV exert a massive impact on the gut microbiome. It has thus been conclusively established that HIV-infected individuals develop an impaired immunity combined with a disorganized intestinal microbiome.

In general, HBV provokes an acute infection in adults. Although most people with healthy immune systems are able to clear the virus, in individuals who have impaired immunity, the initial acute infection may progress to chronic HBV infection. Additionally, it has been demonstrated that immature immune systems and unstable intestinal flora are factors responsible for most chronic HBV cases in infants and young children (5, 108). Thus, the pre-existing immune function and the resident intestinal flora of the host should be considered as critical factors for chronic HBV development, as well as the infective viral load, the virulence of the infective strain, and the invasion pathway of HBV. One study has observed that the intestinal microbiome is an important mediator of the interaction between the intestine and the liver. Indeed, the intestine and the liver share the same embryonic origin, and are linked by the portal venous system. Therefore, the portal vein can be considered to be a physical bridge connecting these structures (109). Knowing that the immunological function and the intestinal flora of the host are determinant factors for HBV infection, it is thus appropriate to address the role of HIV infection in favoring HBV infection establishment.

**Figure 2:**
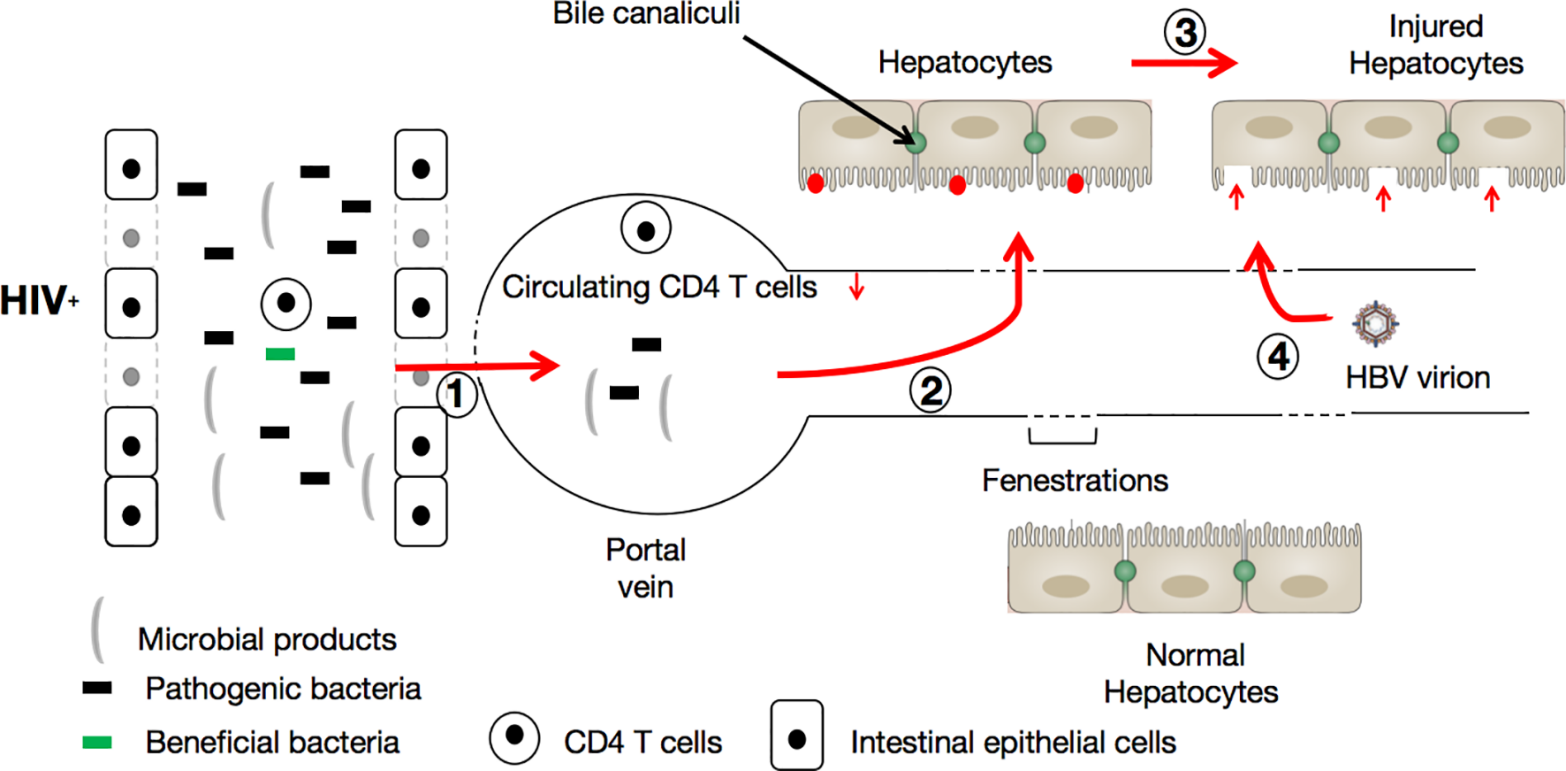
HIV infection facilitates HBV infection *via* the triggering of the leaky gut syndrome. During HIV infection, immune cell (CD4+ T-cells, Th17, and Th22) depletion and bacterial diversity reduction in favor of potentially pathogenic microbe augmentation progressively allows microbes (including pathogenic bacteria) and their products to be translocated (1) into the circulatory system. Here, the translocated microbial products cause further depletion of CD4+ T-cells. Once in the portal vein, the translocated microbes and their products are able to reach hepatocytes (2) and activate the liver’s innate immune system (red points). Consequently, hepatocytes are damaged (3) by pathogen-associated molecular patterns (PAMPs) produced by intestinal microbes, and become vulnerable (red vertical arrows) to HBV incursion and infection (4).

During HIV infection, gut bacterial diversity is diminished while the proportion of potentially pathogenic bacteria is increased (110), and an increased permeability of the intestinal tract is promoted. Consequently, harmful bacteria and their products (LPS for instance) can activate the liver’s innate immune system (via their passage through the portal vein into the liver) by recognition of Toll-like receptors (TLRs, especially TLR2 and TLR4) (111). This natural immune response elicited by pathogen-associated molecular patterns (PAMPs) produced by intestinal microbes could account for the injury to hepatocytes (12). It is known that HBV, upon entering the circulatory system, possesses an extraordinary capacity to specifically infect hepatocytes. The infection of hepatocytes by HBV occurs as follows: (i) HBV attaches to heparan sulfate proteoglycans (HSPGs) that extend from the space of Disse, which separates hepatocytes from liver sinusoidal endothelial cells (LSECs) (112, 113), *via* the engagement of specific loops in the envelope protein, then (ii) the virus penetrates the hepatocyte *via* the interaction of a specific domain of HBV L envelope protein with the sodium taurocholate co-transporting polypeptide (NTCP) (114) on the surface of the hepatocyte. In a context of HIV infection, which causes microbial product translocation and induces hepatocyte injury, the ‘gate is widely open’, so to speak, for HBV to penetrate hepatocytes and initiate the process of viral replication (with an estimated doubling time of 2-4 days) (115, 116) (See **Figure 2**).

It has been reported that the outcome of HBV infection does not strictly depend on the infective viral dose, meaning that even inoculation with a very low HBV dose combined with associated host immunopathology can still result in chronic HBV infection. In the context of HIV, characterized by chronic inflammation and translocated gut microbiota which potentially increases the risk of injury to the liver, active HBV infection has an increased likelihood to eventuate. HBV infection ultimately depends on the relationship between the kinetics of viral spread and the priming of the adaptive immunity, particularly that of HBV-specific CD4+ T-cells (115), which represent essential facilitators of the induction and maintenance of both CD8+ T-cells and antibody responses (117). In addition, HBV-specific CD8+ T-cells can be considered as the ultimate effectors of viral clearance since they are involved in the killing of infected hepatocytes and the local production of cytokines. It has been reported that depletion of CD8+ and/or CD4+ T-cells during acute HBV infection prevents both viral clearance and the onset of liver disease (115, 118). During HIV infection, CD4+ T-cells are depleted, which suggests that finding and recruiting enough CD4+ T-cells to specifically target HBV to prevent subsequent infection represents an onerous task for the immune system. More importantly, studies in chimpanzee (119–121) and human models (122) have revealed that HBV is highly efficient at avoiding recognition by the innate immune system, and seems not to elicit a detectable innate immune response in the chronic HBV context. It is thus valid to hypothesize that HIV infection does greatly favor the establishment of HBV infection *via* its ability to detrimentally modulate both gut microbiota and immune homeostasis.

## Conclusion

HBV infection is recognized to be prevalent among PLWH, and causes poor clinical outcomes in these patients. Based on recent evidence, we speculate that altered gut microbiome composition and diversity, and microbial products (e.g., LPS and SCFA) are involved in systemic immunological impairment in PLWH, and facilitate the subsequent pathogenesis of HBV infection in PLWH. However, more investigations are warranted in the future to precisely elucidate the relationships between microbiota, immunity, HIV, and HBV infection. Collaborative efforts encompassing immunology, microbiology, epidemiology, pharmacology, pathology, and clinical medicine are likely to develop potential strategies (e.g., fecal microbiota transplantation, probiotics, and metformin) to optimize gut microbiota populations and diversity, and to maintain gut homeostasis, thus potentially improving immunological function and preventing secondary viral infections in PLWH.

## Author Contributions

JO and SZ wrote the first draft of the manuscript. XZ, MQ, AH, and HW provided critical revision of the manuscript. YC conceived and designed the manuscript. All authors approved it for publication.

## Funding

This work was supported by the Joint Medical Research Project (2020GDRC010, 2020GDRC004) of Chongqing Science & Technology Bureau and Chongqing Health Commission, Chinese Federation of Public Health Foundation (GWLM202024) and Chongqing Science and Technology commission (cstc2020jscx-cylh0004).

## Conflict of Interest

The authors declare that the research was conducted in the absence of any commercial or financial relationships that could be construed as a potential conflict of interest.

## Publisher’s Note

All claims expressed in this article are solely those of the authors and do not necessarily represent those of their affiliated organizations, or those of the publisher, the editors and the reviewers. Any product that may be evaluated in this article, or claim that may be made by its manufacturer, is not guaranteed or endorsed by the publisher.
